# The Impact of Heterotopic Spleen Regeneration on Tumor Growth

**DOI:** 10.1096/fba.2025-00254

**Published:** 2026-01-09

**Authors:** Daria Artemova, Andrey Elchaninov, Anna Soboleva, Irina Arutyunyan, Polina Vishnyakova, Liudmila Gaydeek, Timur Fatkhudinov, Gennady Sukhikh

**Affiliations:** ^1^ Avtsyn Research Institute of Human Morphology of Federal State Budgetary Scientific Institution “Petrovsky National Research Centre of Surgery” Moscow Russia; ^2^ Research Institute of Molecular and Cellular Medicine, Peoples' Friendship University of Russia (RUDN University) Moscow Russia; ^3^ Petrovsky Medical University Moscow Russia; ^4^ National Medical Research Center for Obstetrics, Gynecology and Perinatology Named After Academician V.I. Kulakov of Ministry of Healthcare of Russian Federation Moscow Russia; ^5^ Department of Histology Cytology and Embryology, Peoples' Friendship University of Russia (RUDN University) Moscow Russia

**Keywords:** heterotopic spleen regeneration, macrophages, mouse model, splenectomy, tumor

## Abstract

The spleen is one of the key organs of the immune system that is involved in both innate and adaptive immunity. Splenectomy (SE) is surgery with many risks, including sepsis, thrombosis, and malignancy. In this regard, studies into the potential regeneration of the spleen and restoring its structure and functions are important. As in the past, heterotopic spleen transplantation regenerates the spleen structure after 30 days. This study assessed the impact of heterotopic spleen regeneration on tumor growth. We used two animal models. In the first, animals had SE. In the second, animals had SE and subcutaneous transplantation (ST) of spleen fragments. These animals, as well as intact animals, were transplanted under the skin with tumor cells to obtain a subcutaneous model of tumor growth. The results showed that while there was no significant effect on tumor growth at 15 days, there was a decrease in tumor cell proliferation rate. Spleen regeneration stimulated early occupancy of the tumor niche by macrophages, as well as influx of CD4+ T‐lymphocytes and B‐lymphocytes into the tumor, while infiltration of CD8+ T‐lymphocytes was suppressed. Thus, the effects of regenerating spleen on tumor growth that we have demonstrated require further investigation at longer follow‐up periods.

## Graphical abstract

The present study evaluated the effect of spleen regeneration on tumor growth. Balb/c mice were divided into three groups: intact animals; animals that underwent splenectomy; animals that underwent splenectomy followed by autologous subcutaneous transplantation of spleen fragments. Then, after 30 days, all animals were injected subcutaneously with LLС‐1 cells and tumor growth was observed. We removed the animals from the experiment on Days 7, 12 and 15, and tumors were taken for further analysis.

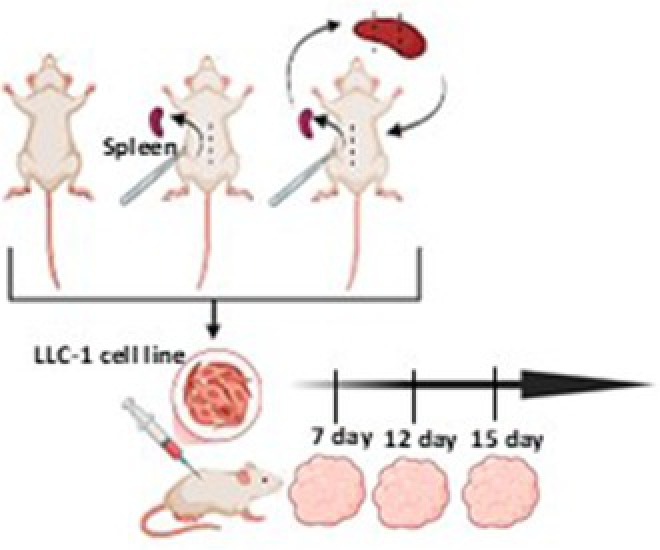

**Abbreviations**
DAPI: 4′,6‐diamidino‐2‐phenylindole
FBS: fetal bovine serum
FITC: fluorescein isothyocyanate
GAPDH: glyceraldehyde 3‐phosphate dehydrogenase
HRP: horseradish peroxidase
LLC‐1: Lewis lung carcinoma
NK: natural killer
PBS: phosphate‐buffered saline
PCR: polymerase chain reaction
SE: splenectomy
SSC: side scatter
ST: subcutaneous transplantation
TAMs: tumor‐associated macrophages
TANs: tumor‐associated neutrophils

## 1 Introduction

The spleen is the largest secondary lymphoid organ. In this organ, old and damaged red blood cells are removed, blood is filtered, and the innate and adaptive immunity systems are activated in response to pathogens entering the body [1]. In addition to its functions under physiological conditions, the spleen plays an important role in tumor growth. Previous studies have shown that the spleen accumulates many hematopoietic stem cells, as well as precursors of macrophages and granulocytes [2, 3]. Monocytes and neutrophils that differentiate from progenitor cells have been shown to migrate into the tumor and become tumor‐associated macrophages (TAMs) and neutrophils (TANs) [2, 3]. Therefore, tumor‐infiltrating TAMs and TANs stimulate tumor invasion and growth and impair patient survival [2, 4].

Furthermore, during the growth of a tumor, monocytes that have migrated from the bone marrow to the spleen present tumor antigens to CD8+ T‐lymphocytes. This contributes to the development of tolerance in these lymphocytes to tumor cells [5]. Thus, the spleen is a key organ in the development of host immune system tolerance to tumor and contributes to tumor progression. Consequently, SE is employed as a therapeutic modality for various malignancies, including ovarian cancer and multiple lymphomas [6, 7].

SE is a surgical procedure in which the spleen is removed. Indications for this procedure include blunt abdominal trauma, which may have resulted in spleen injury; spleen rupture; extensive operations on other abdominal organs; benign and malignant hematologic conditions; and hypersplenism [7, 8]. Given the important role of the spleen in immune response formation, SE is associated with various postoperative complications, with the risk being elevated more than 10‐fold postoperatively. These possible postoperative consequences of SE include infections, thrombosis, and venous thromboembolism [7, 9, 10].

Tumor growth is distinct from other postoperative consequences of SE. While we have previously mentioned the role of the spleen in tumor progression, there are currently different opinions on the effect of SE on tumor progression. On the one hand, experimental animal models have demonstrated a significant decrease in TAMs and TANs post‐SE, both pre‐ and post‐tumor formation. In addition, SE led to a decrease in the rate of tumor progression but did not cause a complete stop of this process [3]. In the following study, the long‐term consequences were analyzed and, more than 20 years after SE, about 13% of the analyzed patients were diagnosed with malignant neoplasms of various genesis [11]. Another study on mouse models of cancer found that SE had no effect on primary tumor growth. However, it could lead to a reduction in metastasis, depending on the pathway involved [12]. In a recent study on a mouse subcutaneous model of colorectal adenocarcinoma, Kaneko et al. demonstrated that SE suppressed primary tumor growth, accompanied by a decrease in TAMs content and activation of T‐cell immunity. However, SE also increased the formation of lung metastases, associated with suppression of natural killer (NK) cell immunity [13]. Therefore, the impact of SE on the growth of tumors may be associated with the sensitivity of the cells of a specific malignancy to various immune system agents [13].

So, SE, regardless of the reason for the operation, is a procedure that is characterized by serious consequences for the body as a whole. Consequently, a promising area of research is the study of potential spleen regeneration and its impact on the body as a whole [14, 15, 16]. The existence of such a condition as splenosis has long been known in the literature, and there is a solid basis for research into possible regeneration and its impact. Splenosis is a benign condition characterized by the growth of splenic tissue in different anatomical parts of the body as a result of splenic rupture or after surgical intervention [14, 17]. Heterotopic transplantation of spleen fragments under the skin has been demonstrated to restore normal organ architecture, B‐lymphocyte population, and monocyte–macrophage system. However, the T‐lymphocyte population requires a longer recovery period [18]. The objective of the present study was to assess the impact of regeneration in heterotopically transplanted spleen fragments on tumor growth.

## 2 Materials and Methods

### 2.1 Animals

Thirty six male BALB/CJLac mice aged 4–8 weeks and weighing 18–20 g were obtained from the nursery of the Andreevka Branch of the Federal State Budgetary Institution “NCBMT” FMBA of Russia. The animals were kept in standard conditions with free access to water and food 12 h day/night. In 2 weeks after acclimatization, animals were introduced into the experiment. Animals were randomly divided into three groups of 12 each: «intact» group—animals that did not undergo surgery; «SE» group—animals that underwent SE; «SE+ST» group—animals that underwent SE followed by autologous ST of spleen fragments. Surgical procedures were performed under injection anesthesia. Mice were injected with a solution containing 1.7 mg/mouse of Zoletil 100 (Virbac, France) and 0.75 mg/mouse Xylanite (Nita‐Pharm, Russia) (calculated per 25 g mouse). This research was conducted in accordance with the United Kingdom. Animals (Scientific Procedures) Act, 1986 and associated guidelines, the European Communities Council Directive 2010/63/EU, and the National Institutes of Health—Office of Laboratory Animal Welfare policies and laws. All experimental protocols were approved by the local ethics committee of FSBSI “Petrovsky National Research Centre of Surgery” (protocol No. 3 of March 23, 2023).

### 2.2 SE Procedure and Subsequent ST

The animals were anesthetized, then a midline laparotomy was performed, the splenic vein and splenic artery were ligated, and the spleen was extracted. Then, the anterior abdominal wall and skin were sutured with continuous sutures, and the suture was treated with 0.05% chlorhexidine bigluconate solution. After that, the animals in the SE group were placed into cages for postoperative recovery. In contrast, the animals in the SE+ST group underwent a modified procedure. Following the suturing of the abdominal wall lateral to the midline, subcutaneous pockets were formed, into which two fragments of autologous spleen were placed on each side. The skin was then sutured with continuous sutures, which were subsequently treated with 0.05% chlorhexidine bigluconate solution. The animals were then permitted to recover from surgery. According to the findings of previous studies, a period of 30 days is required for the spleen to undergo regeneration [18].

### 2.3 LLC1 Cell Line and a Subcutaneous Model of Tumor Growth

Lewis lung carcinoma (LLC1) cell line (Cat. No. 106, BioCollection, Moscow, Russia) was screened to ensure that it was free of contamination and was cultured in complete nutrient medium containing DMEM/F‐12 with glutamine (Paneco, Russia), 10% fetal bovine serum (FBS) (Biowest, Brasil), penicillin–streptomycin (Paneco, Russia), at +37°C and 5% CO_2_ in 150 cm^2^ culture flasks.

Then 30 days after animal surgery, the cells were removed from the flasks using Versen solution (PanEco, Russia) and 0.25% trypsin–EDTA solution; the cells removed from the flasks were centrifuged at 300 g room temperature for 5 min. After centrifugation, cells were counted on a TC20 automatic counter (Bio‐rad, USA). The cells were then resuspended in physiological saline and injected subcutaneously under the scapula of all animals in three groups at an amount of 5 × 10^6^ cells/mouse. Every 2 days, the animals were weighed and tumor size was measured. The animals were removed from the experiment—4 individuals from each group—and the tumors formed were retrieved by anesthesia overdose on Days 7, 12, and 15.

### 2.4 Histological and Immunohistochemical (IHC) Analysis of the Tumors

After tumor extraction, tissue fragments were frozen in liquid nitrogen, then cryosections were made and transferred to Superfrost Plus Microscope Slides (Thermo Fisher Scientific, USA). The slices were stored at −20°C before staining.

Before staining, the slices were washed with phosphate‐buffered saline (PBS) (Paneco, Russia). Some slices were stained with hematoxylin and eosin, and others were stained with the first antibody anti‐CD68 antibody [EPR23917‐164] (ab283654, Abcam, USA) (dilution 1/200) overnight at +4. Slices were then washed with PBS and incubated with the second antibody—goat anti‐rabbit IgG H&L fluorescein isothyocyanate (FITC) (ab97050, Abcam, USA) (dilution 1/200) for 1 h at room temperature. The nuclei were then stained with 4′,6‐diamidino‐2‐phenylindole (DAPI) (0.1 μg/mL). The quantitative analysis of the content of positively stained cells was conducted using the QuPath software (UK) [19].

### 2.5 Real‐Time Polymerase Chain Reaction (PCR) of the Formed Tumors

Total RNA was isolated from tumor fragments using ExtractRNA buffer (Eurogen, Russia). Then the reverse transcription procedure was performed using the Reverse Transcription Reagent Kit with MMLV Revertase (SK021) (Evrogen, Russia). The primer sequences (Forward/Reverse):

- *Gapdh (AGGCCGGTGCTGAGTATGTC/TGCCTGCTTCACCACCTTCT)*
- *Ccna2* (TGTCCTGGATTGGGTCACTGG/TCAGCCTCCGGGCAGTAGA),
- *Ccnd1* (TGTCGGCGCAGTAGCAGA/AAGATACGGAGGGCGCACAG),
- *Ccne1* (TCAGCCTCCGGGCAGTAGA/GTCAGGACCACACTCGGAGG)
- *Il10* (TAAGTGGCAAAGGGGGCGAG/AGGCTGAGCCCCAATGATGT),
- *Cd68* (GGGGCTCTTGGGAACTACAC/GTACCGTCACAACCTCCCTG),
- *Tgfb* (GTACTCTGGCAGTGACCCCG/AACTGCTCCACCTTGGGCTT)

Amplification reactions were performed in triplicate in a DTprime DT96 detection amplifier (DNA‐Technology, Russia). Gene expression levels were quantified using QGENE software (2‐ΔCt method) [20] with normalization to glyceraldehyde 3‐phosphate dehydrogenase (GAPDH).

### 2.6 Western Blot Analysis

Western blot analysis was conducted in accordance with the methodology delineated in a prior study [18]. The following primary antibodies were utilized in the assay: mouse anti‐GAPDH (Cat. № 5G4, clone 4G5, HyTest, Russia) (dilution 1/5000), goat anti‐arginase 1 (sc‐18,351, Santa Cruz, USA) (dilution 1/700), mouse anti‐CD86 (ab213044, Abcam, UK) (dilution 1/2000), rabbit anti‐Ki67 (ab16667, Abcam, UK) (dilution 1/500), rabbit anti‐Cyclin D1 (ab134175, Abcam, UK) (dilution 1/5000), rabbit anti‐Cyclin B2/CCNB2 (ab185622, Abcam, UK) (dilution 1/1000), rabbit anti‐Cyclin A2 (ab181591, Abcam, UK) (dilution 1/700). В качестве вторых антител использовали: goat anti‐rabbit IgG H&L horseradish peroxidase (HRP) (ab6721, Abcam, UK) (dilution 1/5000), goat anti‐mouse IgG H&L (HRP) (ab6789, Abcam, UK) (dilution 1/2000), donkey anti‐goat IgG (H+L) HRP (A15999, Invitrogen, USA) (dilution 1/2000). Membranes were developed with Clarity Western ECL Substrate (Biorad, USA). The signal was detected on a C‐DiGit Blot Scanner (LI‐COR, USA) using Image Studio Acquisition Software (LI‐COR, USA). The sample's protein content was estimated by normalizing to the loading control protein GAPDH.

### 2.7 Phenotyping of Cell Populations in the Tumor

Cells were isolated from the harvested tumor biopsy by mechanical and enzymatic disaggregation. Biopsy specimens were placed in sterile Hank's buffer (Paneco, Russia); the tissue was washed and shredded with a scalpel, then the tissue was mechanically homogenized and incubated in a 0.05% solution of collagenases type 1/4 (Paneco, Russia) for 25 min at 37°C, and the suspension was passed through a 100 μm filter. Erythrocytes in the resulting suspension were lysed with Red Blood Cell Lysis Solution (130‐094‐183, Miltenyi Biotec, Germany) and fixed with 2% paraformaldehyde; after fixation, cells were washed and resuspended in PBS.

To determine the phenotypes of tumor‐infiltrating cells, isolated cells were stained for 1 h at room temperature in the dark with the following panel of anti‐mouse antibodies: CD8a antibody anti‐mouse VioBlue (130‐123‐865, Miltenyi Biotec, Germany), CD3 antibody anti‐mouse FITC, REAfinity (130‐119‐758, Miltenyi Biotec, Germany), F4/80‐PE REAfinity (130‐102‐422, Miltenyi Biotec, Germany), rat anti‐mouse CD4 StarBright Blue 700 (SBB700) (MCA2691SBB700, Biorad, USA), CD45R antibody anti‐mouse PE‐Vio 770 (130‐102‐817, Miltenyi Biotec, Germany), Ly‐6C antibody anti‐mouse APC REAfinity (130‐111‐779, Miltenyi Biotec, Germany). Cell phenotype was determined using a flow fluorimeter MACSQuant Analyzer 10 (Miltenyi Biotec, Germany), and the data were analyzed using FlowJo Software v10.10 (BD Biosciences, USA).

### 2.8 Statistical Analysis

The subsequent statistical processing of the acquired data was performed using the GraphPad Prism 8 software program (Software GraphPad Software, USA). The normality of distribution was determined by the Shapiro–Wilk test. In instances where a normal distribution was confirmed, a one‐way ANOVA for multiple comparisons, followed by a post hoc Tukey's test, was implemented for statistical analysis. Conversely, for cases where the distribution of data was non‐normal or characterized by a limited sample size, a non‐parametric Kruskal–Wallis test was employed for multiple comparisons, with a post hoc Dunn's test serving subsequently. The statistical analysis was conducted under the two‐tailed hypothesis. To assess the statistical significance of observed differences, a 95% confidence interval was established, with a significance level of *p* < 0.05 being considered statistically significant.

## 3 Results

### 3.1 Spleen Regeneration

**FIGURE 1:**
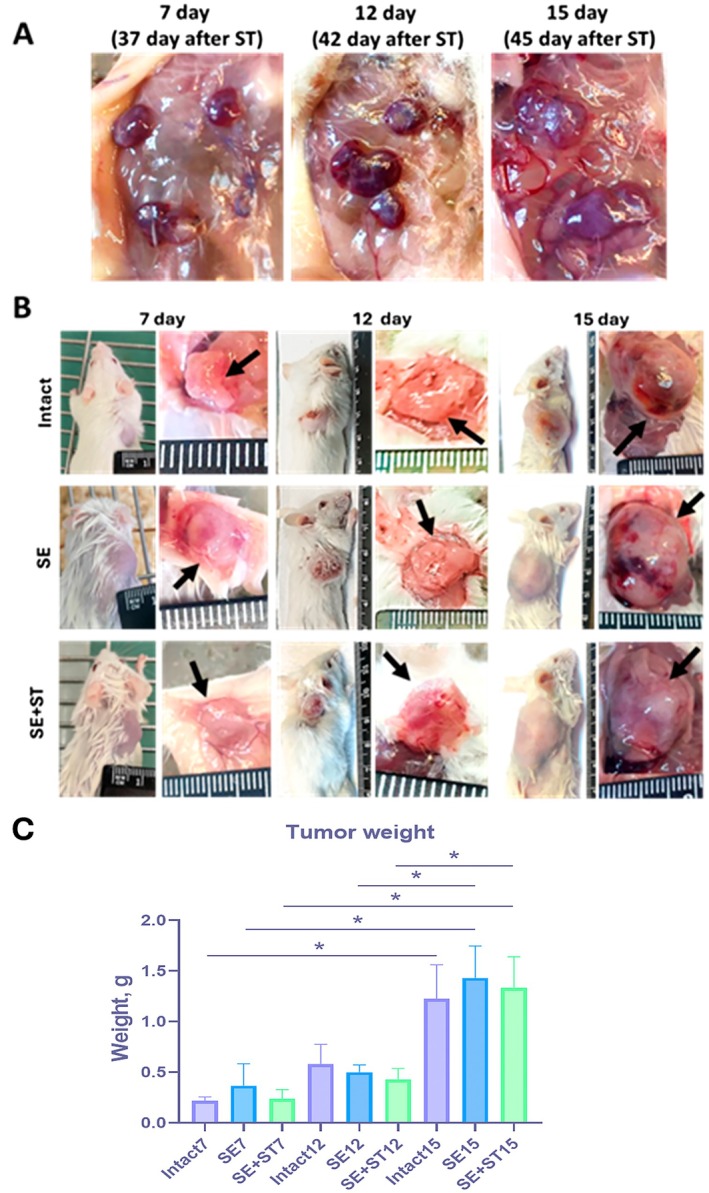
Subcutaneous tumor growth as a result of LLC‐1 cell line transplantation in experimental groups of animals—intact, SE, SE+ST. A, Image of regenerated spleen fragments on 37, 42 and 45 days after surgery. B, Image of subcutaneously formed tumors before autopsy and at autopsy on 7, 12, 15 days after transplantation of LLC‐1 cell line. C, Evaluation of tumor mass on Days 7, 12 and 15 in animals from intact, SE, SE+ST groups. *N* = 4 per group. Statistical analysis was carried out by non‐parametric Kruskal–Wallis test with post hoc Dunn's test (mean with SEM), *р‐value ≤ 0.05, **p‐value ≤ 0.005.

After sacrifice of the animals on 7, 12 and 15 days after transplantation, it was observed that the fragments transplanted under the skin regenerated (Figure 1A). This finding is consistent with the data obtained in a previous study [18].

### 3.2 LLC1 Cells Subcutaneous Transplantation

Seven days after subcutaneous injection of tumor cell line LLC1, tumor growth was observed in all animals in three groups (Figure 1B). The first animals were sacrificed from the experiment on Day 7, and the animals and the extracted tumor were weighed. Then animals were sacrificed on 12 and 15 days after subcutaneous transplantation of tumor cells.

Comparison of tumor masses between groups of animals at each day of sacrifice revealed comparable levels of tumor mass values (Figure 1C). When comparing tumor mass within each experimental group, a significant increase in tumor mass was detected by Day 15. Thus, spleen removal and conversely spleen regeneration had no significant effect on tumor mass on 15 days after tumor cell transplantation, and tumor increased in size over time.

### 3.3 Histologic Architecture of an Established Carcinoma of the Lung

**FIGURE 2:**
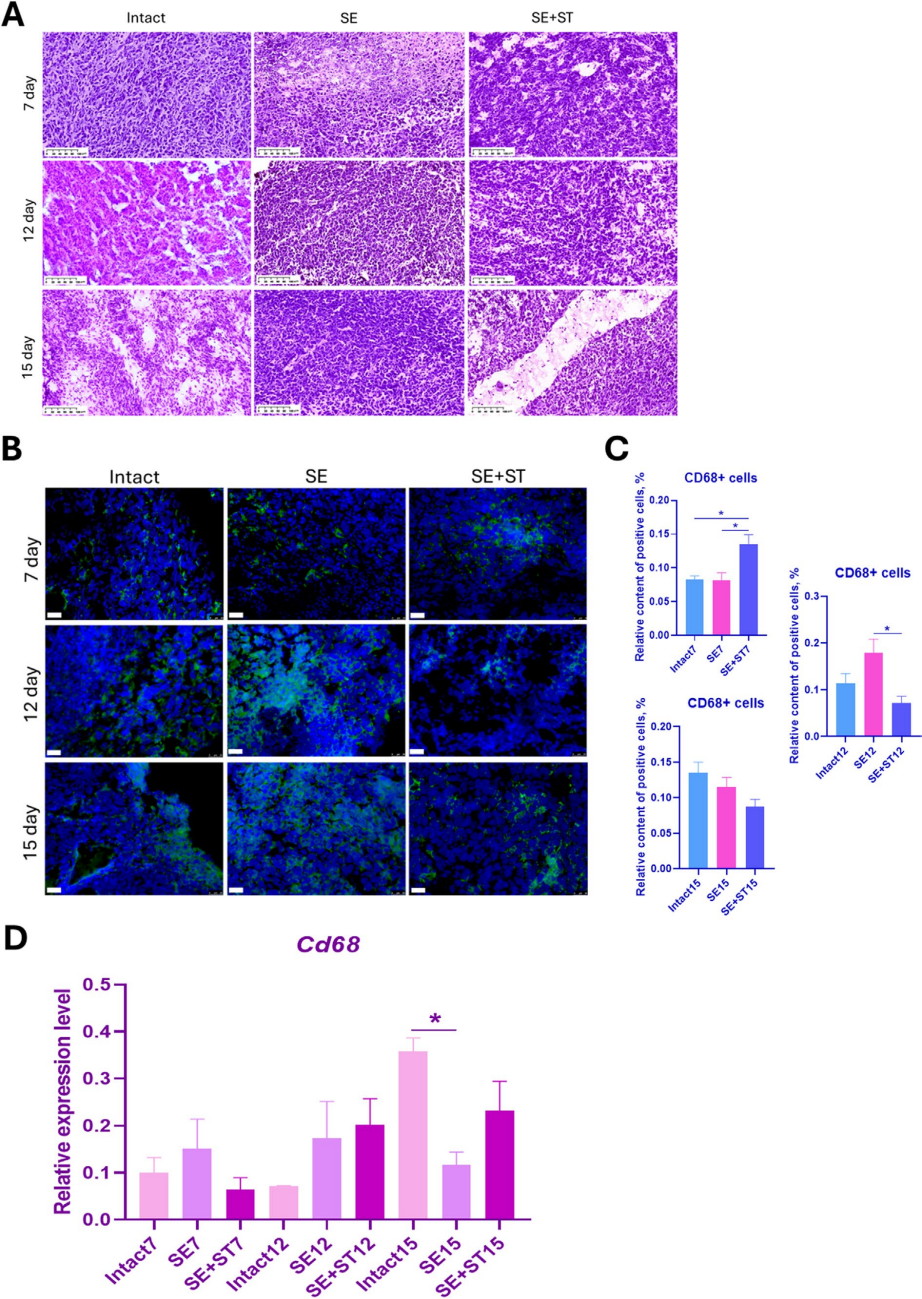
A, histological analysis of tissues of the formed tumors. Hematoxylin–eosin (H&E) staining. B, IHC staining against CD68 (green). Nuclei stained with DAPI (blue). Scale bar 25 μm. C, Relative content of CD68+ cells in formed tumors in animals from intact, SE and SE+ST groups on Days 7, 12 and 15. D, Relative level of CD68 gene expression in the formed tumors of animals from intact, SE and SE+ST groups on Days 7, 12 and 15. The 2^−ΔCt^ method was used to calculate the expression levels, with each gene's expression normalized to the housekeeping gene GAPDH. (B–D) *N* = 3 per group. Statistical analysis was carried out by non‐parametric Kruskal–Wallis test with post hoc Dunn's test (mean with SEM), *р‐value ≤ 0.05.

In histological analysis, we observed densely organized tissue with predominance of cellular content over intercellular substance, and in animals from the SE group, increased deposition of extracellular matrix was observed in some areas of tumors on the 7th day (Figure 2A). The global configuration of tumor structure did not differ significantly between animals of different groups at different time points. In addition, the subcutaneously grown tumors were permeated by blood vessels, which were also detected by histological analysis.

### 3.4 Heterotopic Spleen Regeneration Stimulated Early Recruitment of Macrophages Into the Tumor Microenvironment

In the IHC analysis, we found that 7 days after tumor cell transplantation the content of CD68+ macrophages was significantly higher in animals with regenerated spleen than in other groups of animals. 12 days after transplantation, CD68+ macrophage content was significantly increased in animals that underwent SE compared to other groups. Whereas by Day 15, CD68+ macrophage content in tumors from intact animals was increased, and the differences were not significant. When analyzing the relative level of *Cd68* gene expression, we found non‐significant differences on Days 7 and 12 between the groups (Figure 2D). At the same time, on the 15th day there was a significant increase in *Cd68* gene expression in intact animals, which is consistent with the data of IHC analysis. Thus, spleen regeneration stimulated the recruitment of macrophages into the tumor at early stages of tumor development, while in intact animals the increase of macrophage recruitment into the tumor is observed only 15 days after cell transplantation.

**FIGURE 3:**
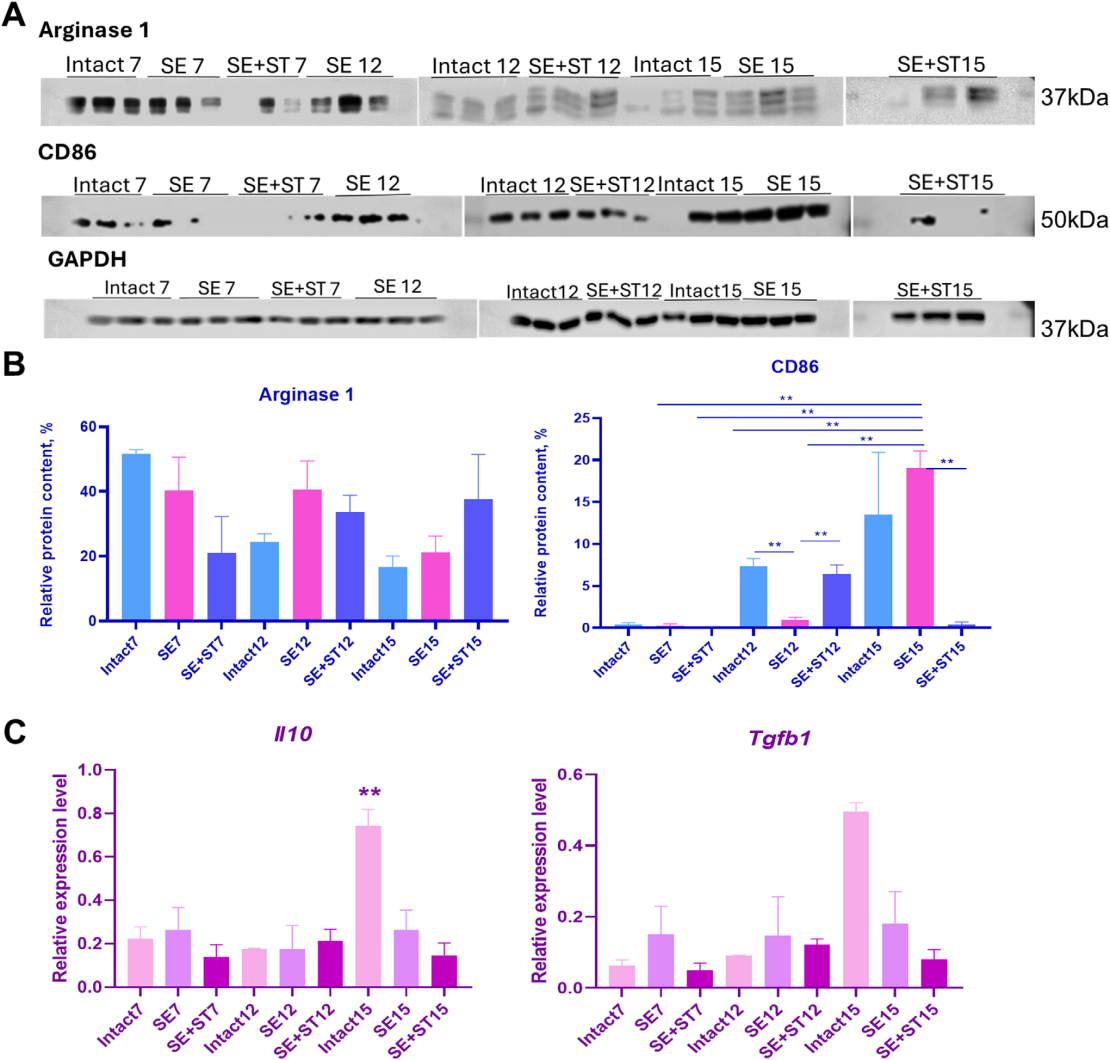
A, Western blot analysis of Arginase 1, CD86 proteins in formed tumors from intact, SE and SE+ST animals at 7, 12 and 15 days. Loading control GAPDH. B, Relative level of analyzed proteins in tumor. C, Relative expression levels of Il10 and Tgfb1 genes in tumors. The 2^−ΔCt^ method was used to calculate the expression levels, with each gene's expression normalized to the housekeeping gene GAPDH. (A–C) *N* = 3 per group. Statistical analysis was carried out by non‐parametric Kruskal–Wallis test with post hoc Dunn's test (mean with SEM), *р‐value ≤ 0.05, **p‐value ≤ 0.005.

Then we analyzed the phenotype of macrophages in the formed tumors. The production of Arginase 1 protein, a marker of macrophages with an anti‐inflammatory phenotype, was observed in tumors from all groups of animals at Days 7, 12, and 15 (Figure 3A). The production of this protein was decreased in tumors from SE+ST animals on Day 7 and gradually increased by Day 15 compared to other groups of animals (Figure 3B). Analyzing the production of CD86 protein, a marker of macrophages with a proinflammatory phenotype, we found a low content of this protein in animals of the three groups at Day 7 compared to Days 12 and 15 (Figure 3A,B). By Day 12 there was an increase in the production of this protein in tumors in intact animals and SE+ST animals; the difference was significant compared to the SE group. But by Day 15 there was a serious increase in the production of this protein in SE animals; the difference was significant in relation to the SE+ST group, as there was practically no production of this protein in tumors in this group by Day 15. Thus, spleen regeneration resulted in macrophages migrating to the tumor acquiring an anti‐inflammatory phenotype at later stages of tumor formation, which was associated with increased production of a proinflammatory polarization marker and decreased production of a proinflammatory polarization marker in tumors by Day 15, compared with intact animals and SE animals. Whereas, when analyzing the relative gene expression levels of anti‐inflammatory cytokines, *Il10* and *Tfb1*, we observed an increase in the expression of these genes in intact mice on Day 15 compared to other groups of animals (Figure 3C). The expression of these genes was lowest in SE+ST animals on Day 15.

### 3.5 Spleen Regeneration Resulted in a Decreased Rate of Cell Proliferation in the Tumor During the Initial Stages of Tumor Growth

**FIGURE 4:**
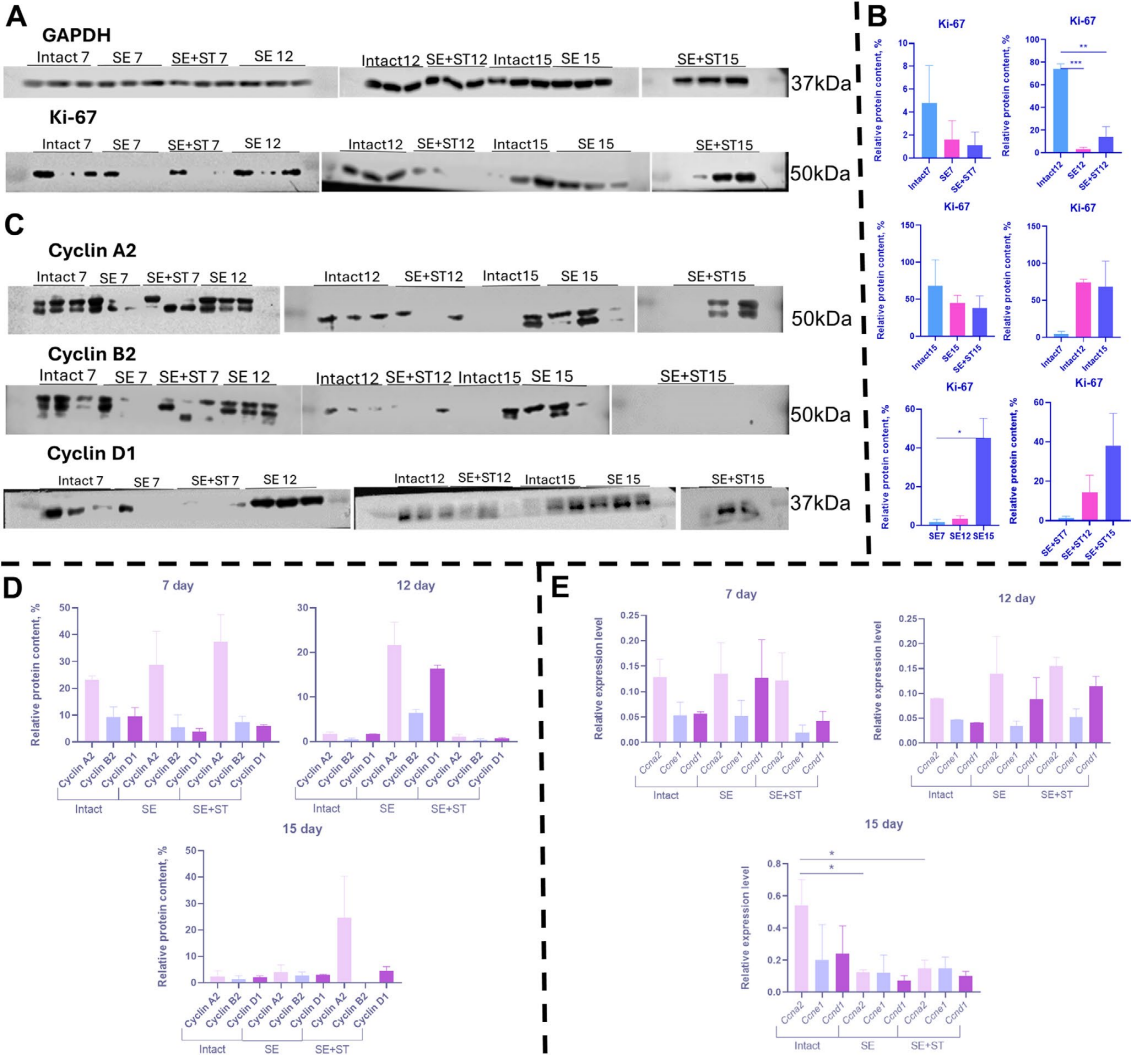
A, Western blot analysis of Ki‐67 protein in formed tumors from intact, SE and SE+ST animals on Days 7, 12 and 15. B, Relative level of Ki‐67 protein in tumors. C, Western blot analysis of Cyclin A2, Cyclin B2 and Cyclin D1 proteins in formed tumors from intact, SE and SE+ST animals on Days 7, 12 and 15. Loading control GAPDH. D, Relative level of Cyclin A2, Cyclin B2 and Cyclin D1 proteins in tumors. E, Relative expression levels of Ccna2, Ccne1, Ccnd1 genes in tumor. The 2^−ΔCt^ method was used to calculate the expression levels, with each gene's expression normalized to the housekeeping gene GAPDH. (A–E) *N* = 3 per group. Statistical analysis was carried out by non‐parametric Kruskal–Wallis test with post hoc Dunn's test (mean with SEM), *р‐value ≤ 0.05, **p‐value ≤ 0.005, ***p‐value ≤ 0.0005.

When analyzing the level of cell proliferation in tumors, we evaluated the level of Ki‐67 protein production (Figure 4A) and evaluated the expression of cyclin genes and their production (Figure 4C,E). Ki‐67 protein production was highest in intact animals when the three experimental groups of animals were analyzed on Days 7, 12, and 15, respectively (Figure 4B). In addition, each group of animals was characterized by an increase in Ki‐67 protein levels on Days 12 and 15 compared to Day 7.

We also found that SE+ST animals showed increased *Ccna2* gene expression and Cyclin A2 protein production in the tumor after 7 days. These levels were comparable to those observed in intact and SE animals (Figure 4D,E). Meanwhile, *Ccnd1* gene expression and synthesis of its product, as well as *Ccne1* gene expression level and Cyclin B2 production were decreased in SE+ST animals. On Day 12, increased production of Cyclins A2, B2, and D1 was observed in SE animals compared with intact and SE+ST animals. On Day 15, there was a significant increase in the expression level of the *Ccna2* gene in tumors from intact animals compared to the expression levels of SE and SE+ST animals. At the same time, Cyclin A2 production was increased in tumors from SE+ST animals compared to other groups of animals. Thus, data from western blot and PCR analysis indicated that on Day 7 comparable levels of proliferation were observed in the three groups of animals, on Day 12 increased proliferation was observed in SE animals. On Day 15, there was increased proliferation in intact animals, as seen in the IHC data on Ki‐67+ cells.

Thus, the greatest proliferation is possessed by cells in the tumor in intact animals. Regardless of the group of animals in each group, the level of Ki‐67 protein increased over time, and SE and spleen regeneration resulted in the fact that at the early stages of tumor growth, the rate of cell proliferation was reduced.

### 3.6 The Role of Spleen Regeneration on the Rate of Tumor Repopulation by Components of the Leukocyte Fraction

**FIGURE 5:**
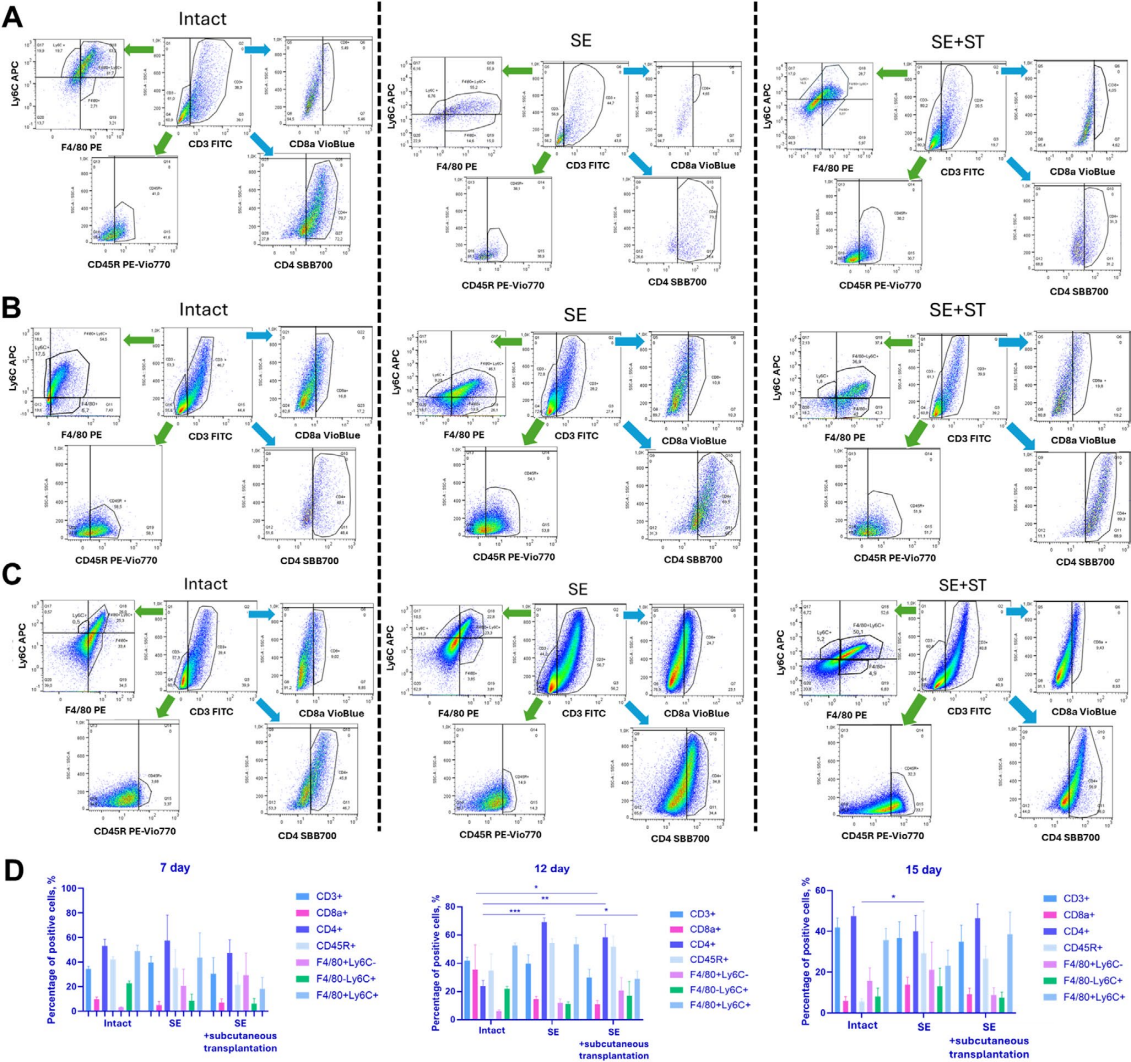
Analysis of cell population in tumor of intact, SE and SE+ST animals on Days 7 (A), 12 (B) and 15 (C). Blue arrows—gating from CD3+ cell population, green arrows—gating from CD3− cell population. D, Percentage content of CD3+, CD8a+, CD4+, CD45R+, F4/80+Ly6C−, F4/80−Ly6C+, F4/80+Ly6C+ cells. (A–D) *N* = 3 per group. Statistical analysis was carried out by non‐parametric Kruskal–Wallis test with post hoc Dunn's test (mean with SEM), *р‐value ≤ 0.05, **p‐value ≤ 0.005, ***p‐value ≤ 0.0005.

Leukocyte migration rates into the forming tumors were analyzed by flow cytofluorimetry. When the total cell population was analyzed on the side scatter (SSC)‐anti‐CD3 dot‐plot, gating was performed to distinguish between CD3+ and CD3‐ cell populations (Figure 5A). Within the CD3+ T‐lymphocyte population, two distinct populations, CD4+ and CD8+ T‐lymphocytes, were identified. In the CD3‐ cell population, a population of CD45R+ B‐lymphocytes and components of the monocyte–macrophage system positive for Ly6C and F4/80 markers were distinguished.

We found that the number of CD8+ T‐lymphocytes increased in the tumor in intact animals on Day 12 compared to Days 7 and 15, which correlated with a decrease in the content of CD4+ T‐lymphocytes on Day 12 (Figure 5A–D). And the content of CD45R+ B‐lymphocytes decreased by Day 15 (Figure 5C). In intact animals, the study noted an increase in F4/80+Ly6C+ macrophages, which are a type of immune cell, derived from monocyte on Days 7, 12 and 15. However, a shift was observed in the ratio of F4/80‐Ly6C+ monocytes, with a decrease by Day 15 and an increase in F4/80+Ly6C‐ macrophages.

When analyzing tumors from SE animals, we observed that SE resulted in reduced recruitment of CD8+ T‐lymphocytes into the tumor by Day 7, while there was a gradual increase in their content by Days 12 and 15 (Figure 5A–C). The highest content in the leukocytic fraction of the tumor was represented by CD4+ T‐lymphocytes, and the content of CD45R+ B‐lymphocytes was increased on Day 12. The content of F4/80+Ly6C+ macrophages increased in the tumor on the 12th day and decreased by the 15th day. However, the level of F4/80−Ly6C+ monocytes was comparable at all time points (Figure 5D). Intact animals showed an increase in the content of F4/80+Ly6C− macrophages by Day 15.

Furthermore, SE+ST animals also demonstrated a decreased CD8+ T‐lymphocyte content at all time points. As seen in the other animal groups, the majority of leukocytes in the tumor were represented by CD4+ T‐lymphocytes (Figure 5A–C). As seen in the SE animals, the content of CD45R+ B‐lymphocytes was also increased on Day 12. The increased content of F4/80+Ly6C+ macrophages was observed in the tumor on the 15th day, and the content of F4/80+Ly6C+ monocytes corresponded to comparable levels on the 7th, 12th and 15th days, as in SE animals. Conversely, the content of F4/80+Ly6C− macrophages decreased by Day 15.

Thus, in comparison with intact animals and SE animals, SE+ST resulted in early tumor repopulation by F4/80+ macrophages, the pool of which was constantly replenished by infiltrating Ly6C+ monocytes. At the same time, SE+ST showed reduced infiltration of CD8+ T‐lymphocytes and slow rates of infiltration by B‐lymphocytes compared to intact and SE animals. Furthermore, spleen regeneration had no significant impact on tumor infiltration by CD4+ T‐lymphocytes.

## 4 Discussion

For over 35 years, research has been conducted on the effects of the spleen on tumor growth and the effects of SE on tumor growth. However, the results of these studies have not provided unequivocal answers [18, 21]. There are many potential factors that influence differing opinions on these issues. These include the time at which SE was performed relative to tumor transplantation; how susceptible the cells of a particular malignancy are to cellular immune agents; the immunogenicity of the malignancy; and the physiological state of the spleen [13, 18, 21, 22]. Despite the absence of consensus on the impact of SE on tumor progression, the other consequences of this operation carry risks that are just as significant, potentially putting patients' health and life at risk [7, 9].

Previously we and other researchers have shown that after heterotopic transplantation of spleen fragments under the skin there is a restoration of its structure and cellular composition [18, 23]. A number of works show the restoration of the main functions of the spleen in the grafts [23]. For instance, it has been demonstrated that transplanted splenic fragments not only successfully engrafted within 2 weeks, but also initiated the clearance of senescent erythrocyte forms [24, 25, 26], restored the neutrophil count, and maintained their level of phagocytic activity [26], also supporting blood rheological properties and platelet count [25]. Furthermore, splenic regeneration remains a subject of significant clinical relevance, particularly since, as mentioned previously, splenectomy can lead to severe postoperative complications, such as infections. Previous studies have indicated that splenic transplantation protected experimental animals from pneumococcal infection by restoring plasma levels of specific IgM and IgG to those observed in non‐splenectomized controls [27, 28, 29, 30]. A restoration of specific antibody titers has also been documented in a number of clinical studies involving splenic autotransplantation [16, 23].

Taking into account that in a number of studies an increase in the incidence of malignant tumors development after splenectomy was found in patients [11, 23], the question of the influence of spleen regenerates on tumor growth is relevant. However, this issue has been insufficiently studied.

In our study, we evaluated how heterotopic spleen regeneration affects the growth of subcutaneous tumors formed as a result of LLC‐1 cell transplantation. Our findings indicate that both SE and heterotopic spleen regeneration had no significant impact on tumor mass. The tumor mass increased over time in all groups of animals. As previously demonstrated in related studies, SE showed no substantial impact on the growth of primary tumors [12]. At the same time, we have shown that regeneration also has no significant effect on the dynamics of tumor tissue growth. When analyzing the rates of tumor niche formation, we revealed that spleen regeneration stimulated an enhanced influx of macrophages into the tumor microenvironment at early stages. The obtained data are consistent with the existing literature on the role of macrophages in tumor development, highlighting their potential as early responders to transformed cells and contributors to tumor formation [31].

In comparison with intact animals and animals after SE, by Day 15 the production of Arginase 1, a marker of anti‐inflammatory macrophages, increased in animals with regenerated spleen, and the production of CD86, a marker of proinflammatory macrophages, was practically absent. It is known that macrophages migrating to the tumor become TAMs and are characterized by anti‐inflammatory phenotype at the late stages of tumor formation, which contributes to the maintenance of tumor growth [32]. As mentioned previously, the spleen serves as a primary reservoir for TAMs precursors. SE has been demonstrated to decrease TAMs levels [3]. Our study revealed increased anti‐inflammatory macrophages in tumors following heterotopic spleen regeneration, compared to animals without spleens and control intact animals.

We observed a time‐dependent increase in tissue mass and cell proliferation in the tumor tissue. Ki‐67 protein production and an increase in tumor mass were seen by Day 15 in all animals. Our western‐blot analysis and real‐time PCR data also revealed lower rates of cell proliferation in animals with regenerated spleens.

In addition to macrophages that populated the tumor at the early stages of its formation and were constantly replenished by incoming Ly6C+ monocytes, we also observed infiltration of the tumor with lymphocytes in animals with regenerated spleens. The predominant lymphocyte type in the tumors was CD4+ T‐lymphocytes, with a notable presence of B‐lymphocytes by Day 12. In contrast, compared to intact animals and animals after SE, the CD8+ T‐lymphocyte content was reduced at all analysis points. Consequently, the cytotoxic T‐lymphocyte response against the tumor was suppressed due to heterotopic spleen regeneration. In an earlier study, Kaneko et al. demonstrated that in SE animals, primary tumors had a decreased content of TAMs with an anti‐inflammatory phenotype and an increased infiltration of CD8+ T‐lymphocytes, which was accompanied with tumor growth restriction [13]. Our research findings on the model of heterotopic spleen regeneration indicate a contrasting outcome. Specifically, regeneration results in an increase in anti‐inflammatory TAMs and a decrease in CD8+ T‐lymphocytes within the tumors. Specifically, we observed an increase in anti‐inflammatory TAMs and a decrease in CD8+ T‐lymphocytes in tumors following spleen regeneration. This is consistent with our findings that a reduced number of CD3+ lymphocytes was found in spleen regenerates after heterotopic transplantation [18].

## 5 Conclusions

Thus, our model shows that regeneration of a key lymphoid organ does not affect tumor weight reduction by 15 days, but tumor cell proliferation rates are reduced. Spleen regeneration also stimulates an early influx of macrophages into the tumor niche and their constant replenishment by incoming monocytes. The tumor niche in these animals also contained T‐ and B‐lymphocytes, while the content of antitumor cytotoxic CD8+ cells was lower compared with intact and SE animals. To understand the detailed impact of spleen regeneration on tumor growth, the observation period of the animals must be extended. We hypothesize that autologous spleen transplantation, in general, contributes to the potential inhibition of tumor growth at the early stages of its development by facilitating the early recruitment of macrophages into the tumor microenvironment.

## Author Contributions

**Daria Artemova:** methodology, validation, formal analysis, investigation, writing – original draft, writing – review and editing, visualization, funding acquisition. **Andrey Elchaninov:** conceptualization, methodology, resources, writing – original draft, writing – review and editing, supervision, project administration. **Anna Soboleva:** investigation, writing – original draft, writing – review and editing. **Irina Arutyunyan:** investigation, writing – original draft, writing – review and editing. **Polina Vishnyakova:** conceptualization, writing – review and editing. **Liudmila Gaydeek:** investigation. **Timur Fatkhudinov:** conceptualization, writing – review and editing, project administration. **Gennady Sukhikh:** conceptualization, writing – review and editing.

## Funding

This work was supported by Russian Science Foundation [grant number 24‐25‐00138] to D.A.

## Conflicts of Interest

The authors declare no conflicts of interest.

## Supporting information


Data S1.

## Data Availability

The data associated with our study have not been deposited into a publicly available repository. The datasets supporting the conclusions of this article are included within the article and its Supporting Information.
