# Supplementary figures and images for: The Impact of Heterotopic Spleen Regeneration on Tumor Growth
Source: FASEB Bioadv. 2026 Jan 9;8(1):e70076. doi: 10.1096/fba.2025-00254 (PMC12784169; doi:10.1096/fba.2025-00254)

GAPDH


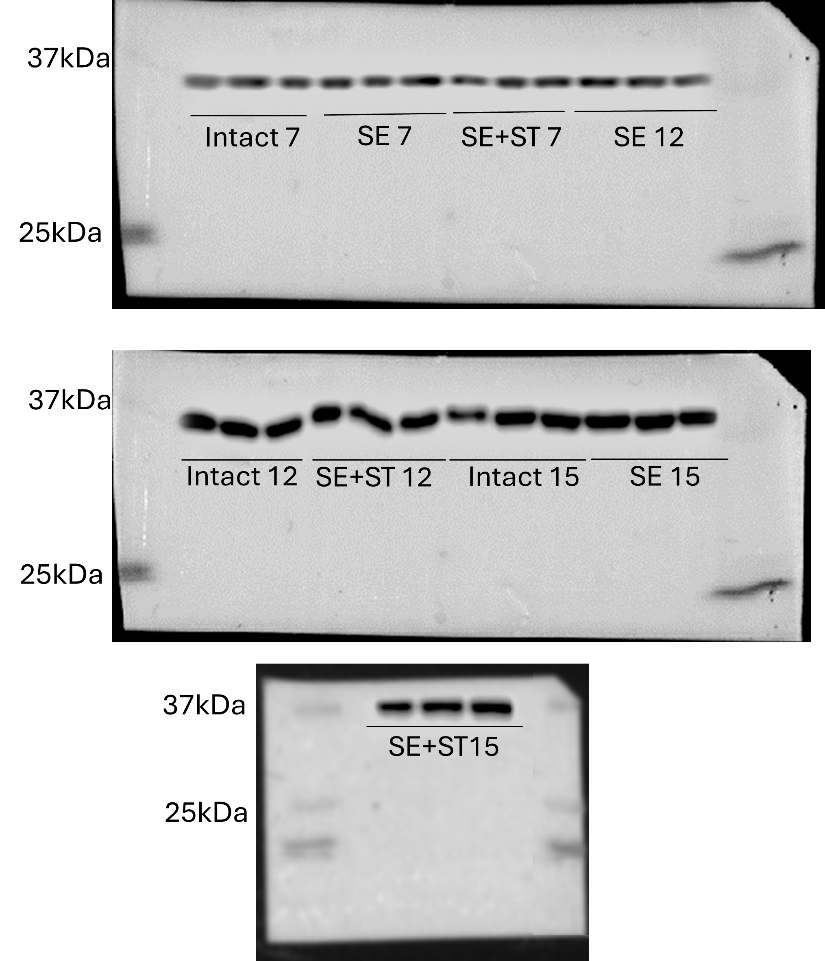


Arginase 1


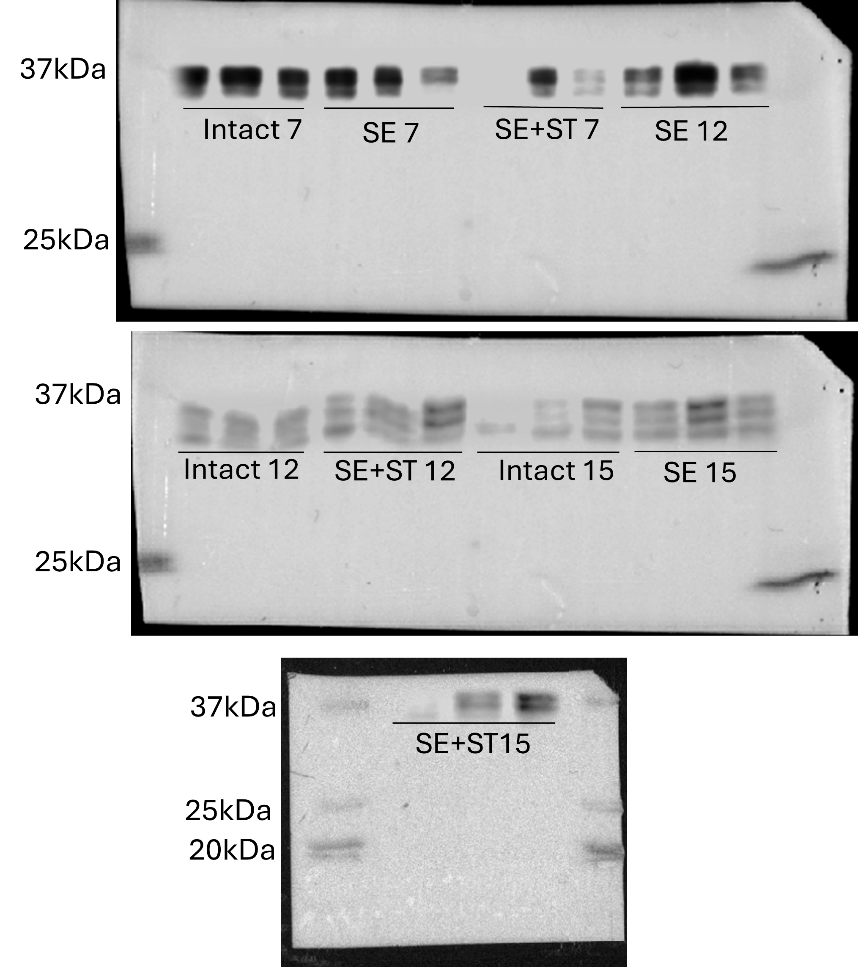


CD86


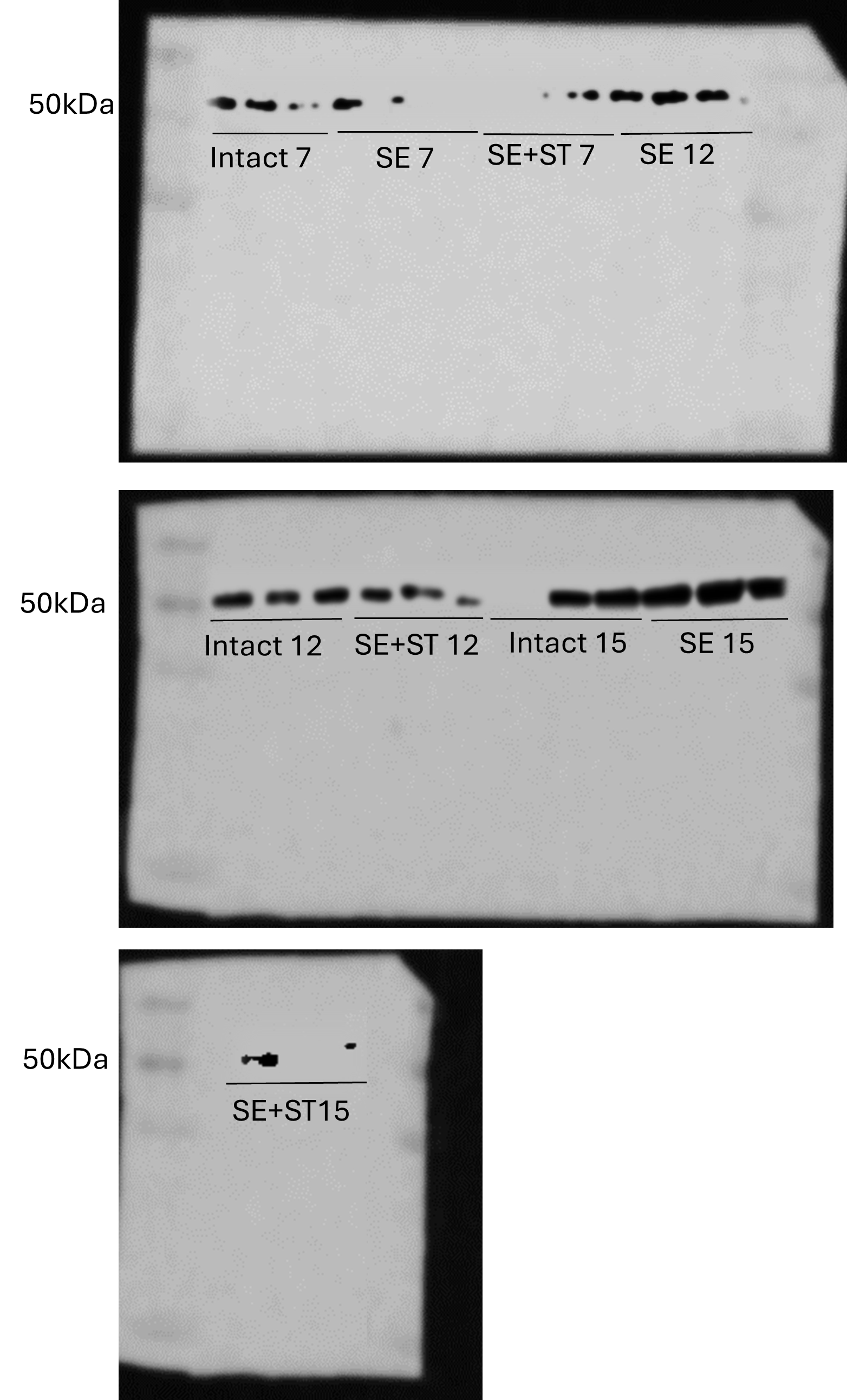


Ki-67


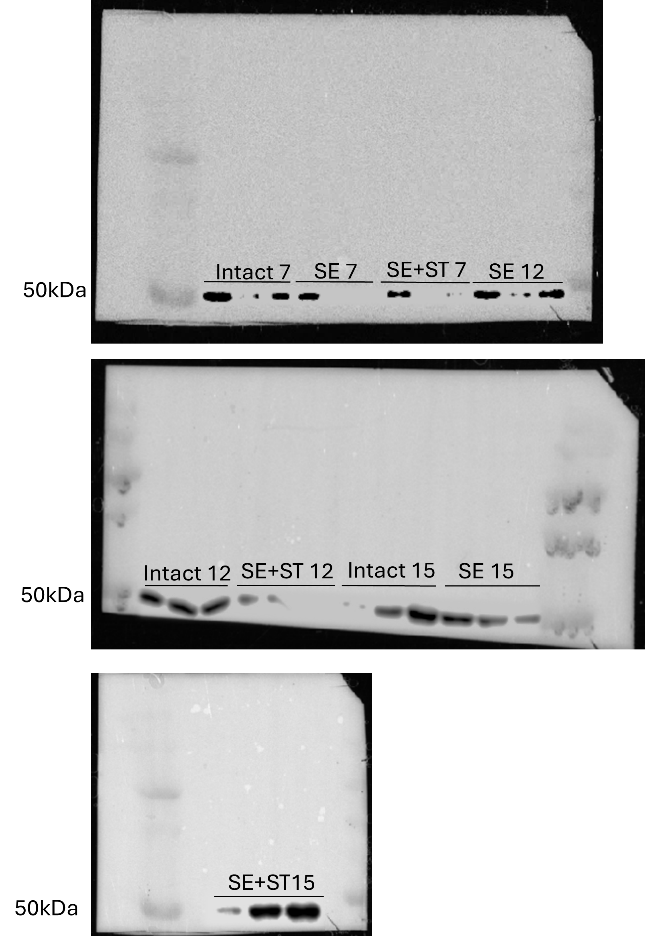


Cyclin A2


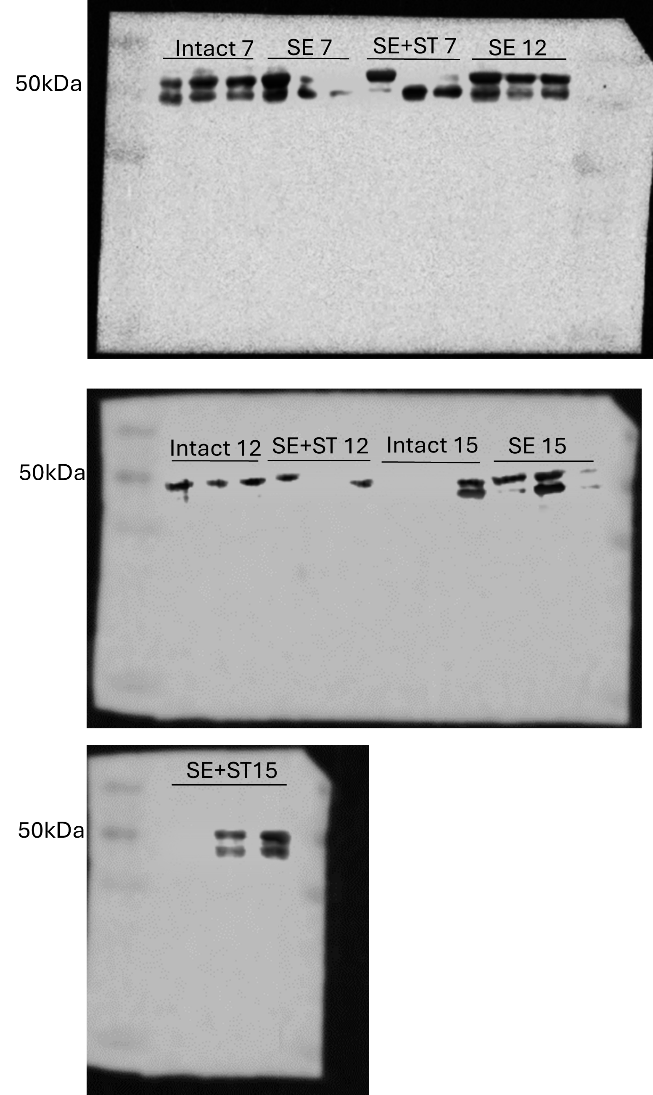


Cyclin B2


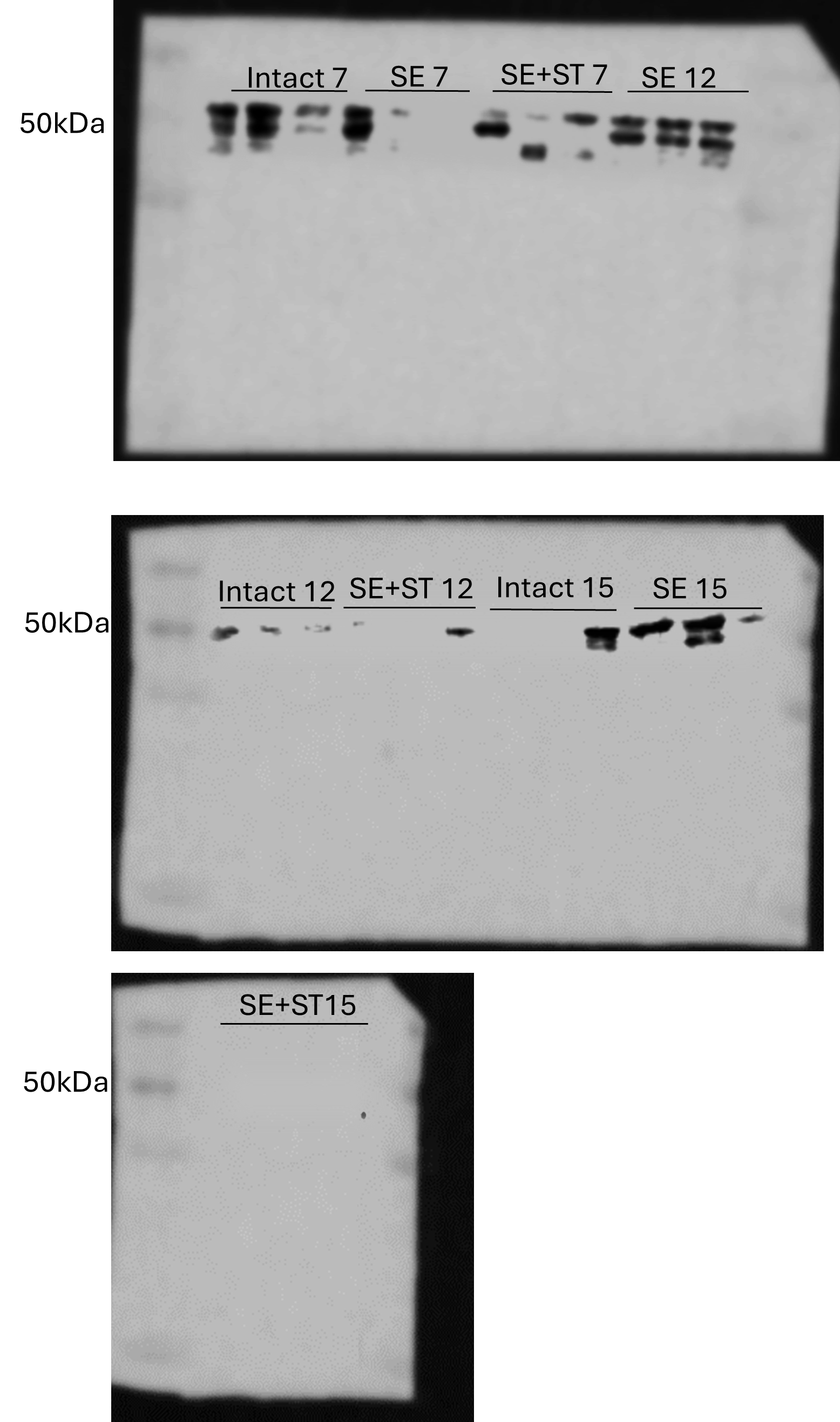


Cyclin D1


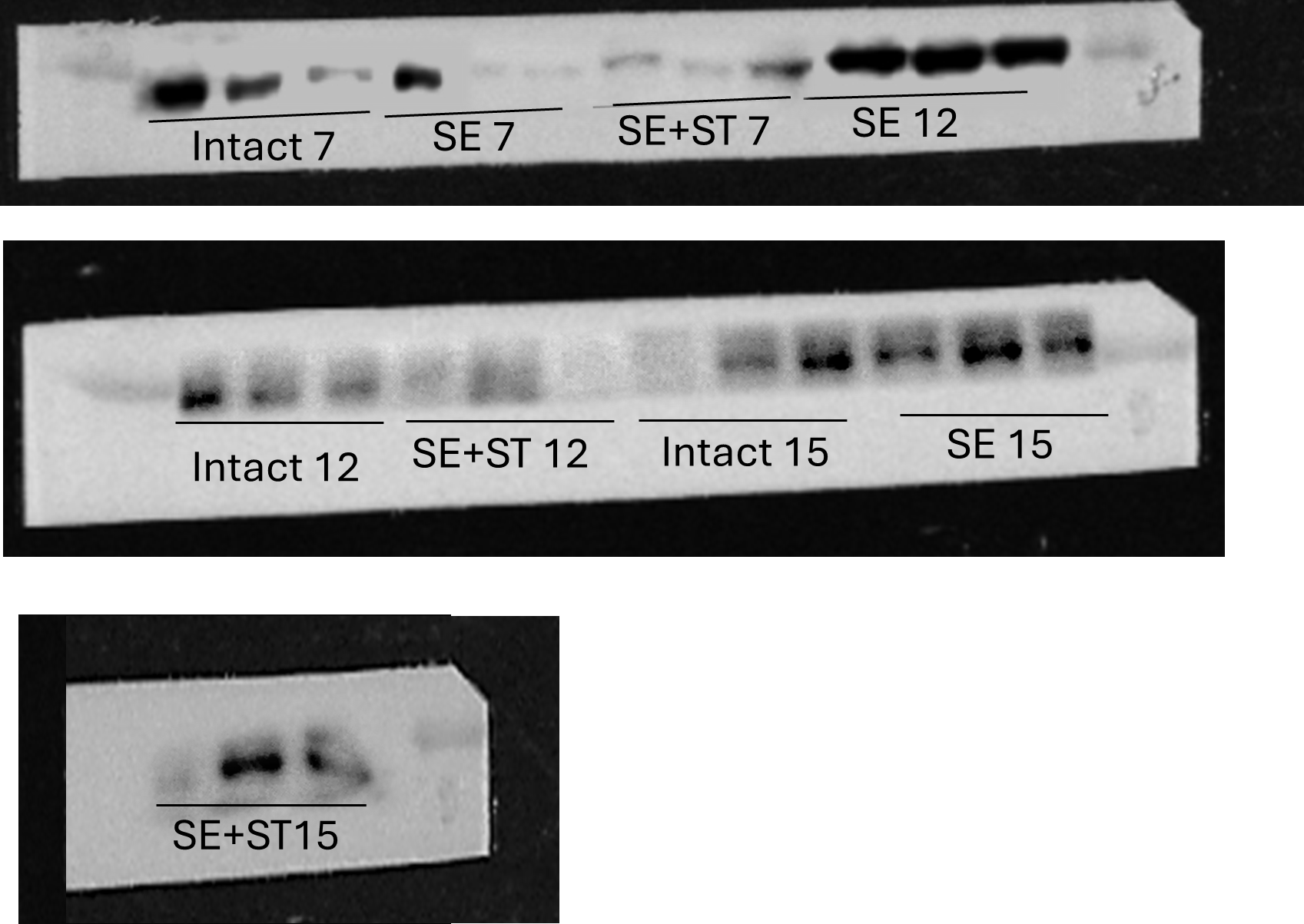

Supplement: Supplementary file 1 — Data S1. [file FBA2-8-e70076-s001.docx]
